# Is hemithyroidectomy enough? Low risk of occult contralateral disease in sporadic medullary thyroid cancer

**DOI:** 10.1007/s00405-025-09929-1

**Published:** 2025-12-22

**Authors:** Andreas Spörlein, Christoph Becker, Joseph Berthelot, Valentin Burkhardt, Katharina Laubner, Mira Fink, Bernd Jänigen

**Affiliations:** 1 Department of Otorhinolaryngology - Head and Neck Surgery, Medical Center - University of Freiburg, Freiburg, Germany; 2 Division of Endocrinology and Diabetology, Department of Medicine II, Medical Center – University of Freiburg, Faculty of Medicine, University of Freiburg, Freiburg, Germany; 3 Department of General and Visceral Surgery, Medical Center - University of Freiburg, Freiburg, Germany

**Keywords:** Medullary thyroid carcinoma, Prognostic factors, Personalized treatment, Risk-adapted surgery, Thyroidectomy, Quality of life

## Abstract

**Purpose:**

Total thyroidectomy (TT) remains the standard surgical treatment of sporadic medullary thyroid cancer (sMTC), primarily due to the presumed risk of occult contralateral disease. However, recent evidence suggests that in selected patients, hemithyroidectomy (HT) may offer comparable oncologic outcomes. This retrospective study aimed to evaluate the prevalence of occult contralateral disease and to identify clinicopathological factors associated with disease-free survival (DFS).

**Methods:**

We retrospectively analyzed all surgical patients with sMTC at a tertiary academic center (2013–2025), excluding patients with hereditary MTC, RET mutations, or clinical evidence of MEN2. Kaplan-Meier analysis with logrank testing was used to identify predictors of DFS.

**Results:**

Forty-eight patients were included (66.7% female, median age 60.5 years). TT was performed in 44 patients (91.7%). Bilateral disease was present in five patients (11.4%), all detected on preoperative ultrasound. No cases of occult contralateral disease were found. During a median follow-up of 3.4 years, seven patients (14.6%) experienced recurrence. Advanced T stage, nodal involvement, distant metastases, lymphatic and vascular invasion, extracapsular extension, and male sex were all significantly associated with reduced DFS (all *p* < 0.05). Persistent postoperative calcitonin elevation (> 2 pg/mL) strongly predicted recurrence (*p* < 0.001), whereas baseline calcitonin > 500 pg/mL did not.

**Conclusion:**

Occult contralateral disease in sMTC is rare when high-quality preoperative ultrasound is available. In selected patients with unifocal, node-negative tumors and favorable pathology, HT combined with structured calcitonin monitoring may be an oncologically safe, less morbid alternative. These findings support individualized, risk-adapted surgical strategies in sMTC. Prospective validation is warranted.

## Introduction

Medullary thyroid carcinoma (MTC) is a neuroendocrine malignancy originating from the parafollicular C-cells of the thyroid gland. It is rare with an incidence below 1 per 100,000, accounting for approximately 3–5% of all thyroid cancers [1, 2]. MTC is distinct in its molecular pathogenesis, clinical course, and therapeutic considerations, when compared to differentiated thyroid carcinomas (DTC) [2, 3]. It arises sporadically in 75–80% of cases, while the remaining are associated with germline RET proto-oncogene mutations in hereditary syndromes such as multiple endocrine neoplasia type 2 (MEN2) [4–6].

In contrast to differentiated thyroid carcinomas, MTC lacks the ability to concentrate radioactive iodine, making complete surgical resection the cornerstone of curative treatment [2, 7]. Historically, total thyroidectomy (TT) with central lymph node dissection has been universally advocated as the standard of care for patients with either confirmed or suspected MTC, based on the tumor’s potential for multifocality, bilateral occurrence, and lymphatic spread.

Most international guidelines continue to endorse TT as the primary surgical approach for patients with sporadic MTC. The American Thyroid Association (ATA) recommends TT with prophylactic central lymph node dissection in all patients with known MTC, regardless of tumor size. Completion thyroidectomy following hemithyroidectomy (HT) is recommended in patients with a RET germline mutation, an elevated post-operative serum calcitonin level, or imaging studies indicating residual tumor [8]. The National Comprehensive Cancer Network (NCCN) [9], the British Thyroid Association (BTA) [10], and the German guideline on thyroid cancer [11] reaffirm this position, stating that TT is the preferred approach for operable cases of MTC. Similarly, the European Society for Medical Oncology (ESMO) emphasizes TT as the standard of care, with the extent of neck dissection guided by preoperative calcitonin levels and imaging findings [12].

As assessed by a recent systematic review, the guideline recommendations for TT are based on low-quality data, mostly single-center retrospective cohort studies [13]. The Japan Associations of Endocrine Surgeons (JAES) states a weak recommendation for HT for sporadic, unilateral MTC [14], quoting a prospective Japanese study from 2003 demonstrating that HT with systematic central neck dissection yielded biochemical cure in 80% of patients with nonhereditary MTC, with no apparent benefit from more extensive surgery [15].

As the rate of complications including postoperative hypoparathyroidism [16, 17] and vocal fold paralysis [18] is higher in TT compared to HT, it is important to consider whether the extent of surgery is beneficial to survival in MTCs. Preoperative ultrasound has become more accurate in recent years, making contralateral occult disease less likely [19, 20].

Emerging evidence suggests that a subset of patients with sporadic, unifocal, low-volume MTC confined to one lobe without evidence of RET mutation, nodal involvement, or severely elevated calcitonin may be candidates for a less extensive approach [21–25]. HT with appropriate nodal staging may represent a safe and effective alternative to TT for selected patients, also reflecting a broader trend in oncologic surgery toward individualized, risk-adapted treatment strategies that balance oncologic control with quality of life [26]. In this retrospective study, we analyzed the risk of occult contralateral disease of sMTCs in patients who underwent thyroid surgery in our tertiary center. Additionally, clinical and pathological risk factors associated with an adverse outcome of disease-free survival were assessed.

## Methods

### Study design and patient selection

A retrospective cohort study was conducted at a tertiary academic medical center. Surgical patients between January 2013 and January 2025 with histologically confirmed MTC were included. Those with incomplete medical records, RET germline mutation, family history or clinical features of multiple endocrine neoplasia type 2 (MEN2) or familial medullary thyroid carcinoma (FMTC) were excluded. All patients underwent surgery performed by experienced endocrine or head and neck surgeons in accordance with current international guidelines. Follow up was performed every 3 months initially and routinely consisted of ultrasound, lab testing of calcitonin, CEA, TSH, fT3, fT4. Additional diagnostics such as calcium stimulation test, MRI, PET/CT, or bone scintigraphy were performed on selected patients.

### Data collection

Clinical, biochemical, radiological, surgical, and pathological data were extracted from electronic health records. Preoperative variables included patient age, sex, serum calcitonin levels, and findings from neck ultrasound. Operative reports were reviewed to determine the extent of thyroidectomy and lymph node dissection. Pathology reports were analyzed for tumor size, focality, TNM classification (AJCC/UICC 8th edition, 2017; older cases were reclassified accordingly), lymphovascular and perineural invasion, and presence of extracapsular extension, and desmoplasia. Follow-up data were collected until the last clinical visit or death.

### Statistical analysis

Descriptive statistics were used to summarize patient and tumor characteristics. Continuous variables were presented as median with interquartile range (IQR), and categorical variables as absolute numbers and percentages. The 5-year disease-free survival rate (5y-DFS) was reported and Kaplan-Meier survival analysis was performed to assess disease-free survival (DFS), defined as the time from initial surgery to first structural recurrence or death from any cause, whichever occurred first. Comparisons between subgroups were conducted using the logrank (Mantel-Cox) test. In an exploratory analysis, the frequency of any and of lateral lymph node metastases between patients with unilateral and bilateral disease were compared using Fisher’s exact test. A p-value of < 0.05 was considered statistically significant. Statistical analyses were performed using GraphPad Prism version 10.5.0 (GraphPad Software, San Diego, CA, USA).

## Results

### Patient characteristics

A total of 48 patients with sporadic medullary thyroid cancer (sMTC) were included in the analysis (Table 1). The cohort consisted of 32 females (66.7%) and 16 males (33.3%), with a median age at surgery of 60.5 years (interquartile range, IQR: 45.3–65.8 years). Most patients presented with early T-stage tumors: T1 in 31 patients (64.6%) and T2 in 10 patients (20.8%). Advanced T-stage disease (T3-T4) was less common, occurring in 7 patients (14.6%). At the time of surgery, the majority of 36 patients (75.0%) were node-negative (N0) while 3 patients (6.3%) were N1a and 9 patients (18.8%) were N1b. Distant metastases were present in 4 patients (8.3%).

**Table 1:** Cohort characteristics

Total (*n*, %)	48 (100)
Gender (n, %)	
Female	32 (66.7)
Male	16 (33.3)
Age at surgery (median, IQR), years	60.5 (45.3–65.8)
Follow up (median, IQR), years	3.4 (1.5–7.7)
T stage (n, %)	
T1	31 (64.6)
T2	10 (20.8)
T3	6 (12.5)
T4	1 (2.1)
N (n, %)	
N0	36 (75.0)
N1a	3 (6.3)
N1b	9 (18.8)
M (n, %)	
M0	44 (91.7)
M1	4 (8.3)
Primary tumor size (median, IQR), mm	14.0 (8.3–27.5)
≤ 20	30 (62.5)
>20 ≤ 40	12 (25.0)
>40	6 (12.5)
Extent of thyroidectomy (n, %)	
Hemithyroidectomy	2 (4.2)
Completion hemithyroidectomy	2 (4.2)
Total thyroidectomy	44 (91.7)
Extent of neck dissection (n, %)	
No neck dissection	3 (6.3)
Central	16 (33.3)
Central and ipsilateral lateral	1 (2.1)
Central and bilateral lateral	24 (50.0)
Central, bilateral lateral and mediastinal	1 (2.1)
Preoperative calcitonin (median, IQR), pg/mL	582.5 (191.3–1362.3)
≤ 500	18 (40.0)
>500	27 (60.0)
Disease recurrence (n, %)	
No	41 (85.4)
Yes	7 (14.6)
Vital status (n, %)	
Alive	42 (87.5)
Dead	6 (12.5)

The median primary tumor size was 14.0 mm (IQR: 8.3–27.5 mm), with 62.5% of tumors measuring ≤ 20 mm. Neck dissection was carried out in the majority of cases, most often comprising the central compartment (level VI) in 33.3% and the central and bilateral lateral compartment (levels II to VI) in 50.0% of cases. The median preoperative calcitonin level was 582.5 (IQR: 191.3-1362.3) pg/mL. During a median follow-up of 3.4 (IQR: 1.5–7.7) years, disease recurrence was observed in 7 patients (14.6%) and 6 patients (12.5%) had died of all causes.

### Identification of risk factors

**Fig. 1:**
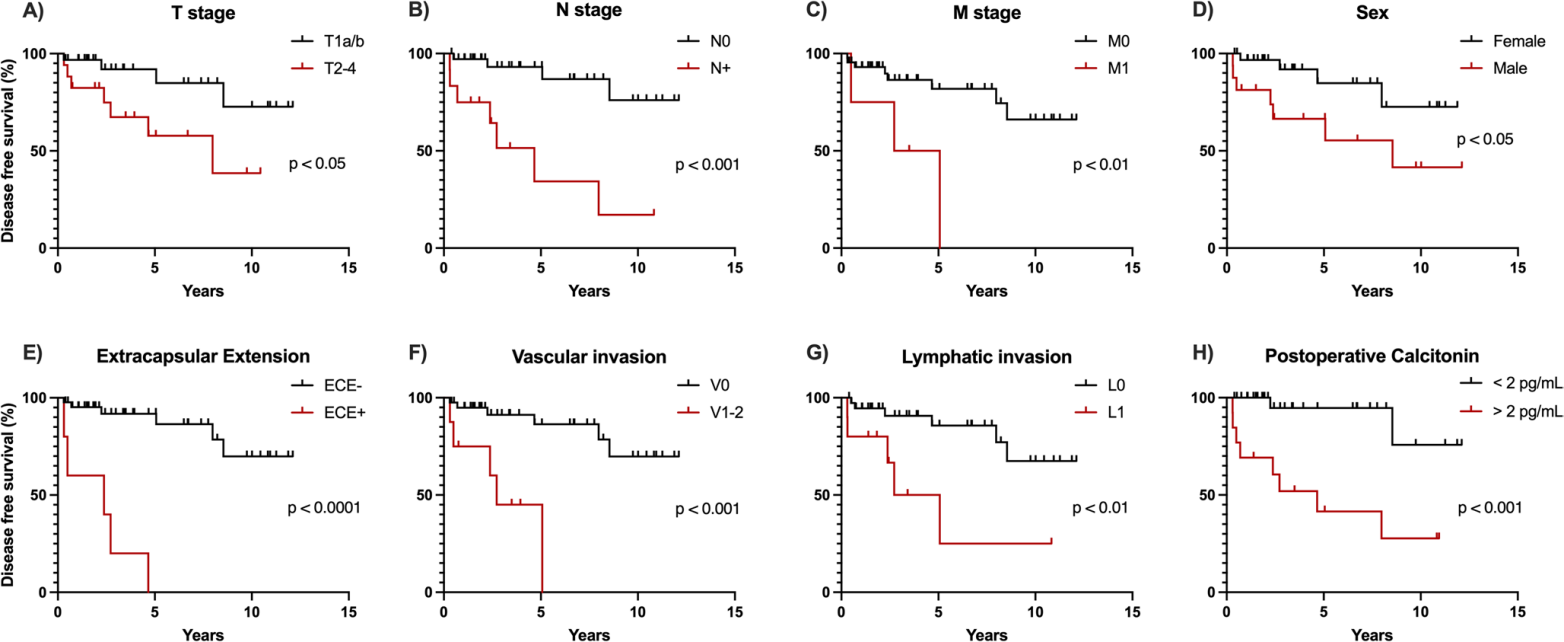
Kaplan–Meier curves for disease-free survival (DFS) in patients with sporadic medullary thyroid carcinoma. DFS was defined as time from initial surgery to first structural recurrence or death from any cause. Curves are shown stratified by (**A**) T stage (T1 vs. T2–T4), (**B**) nodal status (N0 vs. N+), (**C**) presence of distant metastasis (M0 vs. M1), (**D**) sex (female vs. male), (**E**) extracapsular extension (ECE– vs. ECE+), (**F**) vascular invasion (V0 vs. V1–2), (**G**) lymphatic invasion (L0 vs. L1), and (**H**) postoperative basal calcitonin ≥ 2 months after surgery (< 2 pg/mL vs. >2 pg/mL). Censored observations are indicated by tick marks

Kaplan-Meier analysis with logrank (Mantel-Cox) testing revealed several factors significantly associated with adverse outcome (Fig. 1; Table 2). Patients with higher T stages (T2–T4) had significantly lower 5y-DFS compared to those with T1a/T1b tumors (57.8% vs. 91.9%, *p* < 0.05). Similarly, nodal involvement (N1a/N1b vs. N0) was associated with a markedly reduced 5y-DFS (34.3% vs. 93.1%, *p* < 0.001). Patients presenting with distant metastases at diagnosis also had inferior outcomes (50.0% vs. 81.9%, *p* < 0.01).

**Table 2:** Risk factors and their effects on 5-year disease-free survival (5y-DFS)

	5y-DFS	HR (95% CI)	*p*
T2-4 vs. T1	57.8% vs. 91.9%	3.63 (1.03–12.81)	0.027
N + vs. N0	34.3% vs. 93.1%	7.26 (1.63–32.32)	0.0002
M1 vs. M0	50.0% vs. 81.9%	5.20 (0.49–55.05)	0.006
L1 vs. L0	25.0% vs. 85.7%	4.50 (0.88–22.89)	0.006
V1-2 vs. V0	45.0% vs. 86.4%	6.73 (1.02–44.46)	0.0002
ECE + vs. ECE-	0% vs. 91.8%	12.5 (1.09–143.70)	< 0.0001
Male vs. female	66.5% vs. 84.7%	3.38 (0.97–11.77)	0.038
Postoperative calcitonin			
>2pg/mL vs. < 2 pg/mL	41.5% vs. 94.7%	9.76 (2.49 to 38.19)	0.0004
Baseline calcitonin			
>500 pg/mL vs. < 500 pg/mL	86.28 vs. 77.95%	1.63 (0.47–5.77)	0.469

*HR* hazard ratio, *ECE* extracapsular extension, *T, N, M* parameters according to AJCC/UICC 8th edition, *V* vascular invasion, *L* lymphatic invasion

Pathological indicators including lymphatic invasion (L1 vs. L0, 25.0% vs. 85.7%, *p* < 0.01), vascular invasion (V1–2 vs. V0, 45.0% vs. 86.4%, *p* < 0.001), and extracapsular extension (ECE + vs. ECE–, 0% vs. 91.8%, *p* < 0.0001) were all significantly associated with reduced 5y-DFS. Perineural invasion was not observed in any case.

There was no significant difference in 5y-DFS between patients with high (> 500 pg/mL) and low (≤ 500 pg/mL) baseline calcitonin levels (*p* = 0.52). However, patients with persistent calcitonin elevation (> 2 pg/mL) from two months postoperatively had worse outcomes compared to those with undetectable levels (41.5% vs. 94.7%, *p* < 0.001). Male patients showed lower 5y-DFS compared to female patients (66.5% vs. 84.7%, *p* < 0.05).

### Risk of occult contralateral disease

Out of 48 patients, 44 (91.7%) were treated with TT. Five of those patients who received TT (11.4%) had bilateral disease. All patients with bilateral disease were diagnosed on preoperative ultrasound; thus, no occult disease was detected incidentally by resection of the contralateral lobe. Two patients had previously undergone HT for unrelated benign conditions (goiter or thyroid nodule) and were treated with completion thyroidectomy. Two received HT initially and a decision against a secondary completion thyroidectomy was made due to biochemical cure with undetectable calcitonin postoperatively. No recurrence occurred to these two patients until the end of follow up after 1.8 and 8.2 years respectively. Among patients with bilateral disease, 2/5 had lateral lymph node metastases (N1b) and 3/5 had no lymph node metastases (N0). Compared with patients with unilateral disease (lateral lymph node metastases 6/39; any lymph node metastases 9/39), there were no significant differences.

## Discussion

In this retrospective cohort study, we investigated the prevalence of occult contralateral disease in patients with sporadic medullary thyroid carcinoma (sMTC) and analyzed prognostic factors associated with disease-free survival (DFS). Our findings underscore the low rate of clinically occult bilateral disease and provide further support for risk-adapted surgical strategies in selected patients with sMTC. The current international standard for the surgical management of sMTC remains TT with central neck dissection, as endorsed by major guidelines. The American Thyroid Association [8], the National Comprehensive Cancer Network [9], the British Thyroid Association [10], the German guideline on thyroid cancer [11], and the European Society for Medical Oncology [12] recommend TT regardless of tumor size based on low-quality evidence, relying mostly on retrospective, single-center studies [13].

Our cohort showed a low rate of bilateral disease (11.4%), and importantly, all instances were identified preoperatively by ultrasound. No cases of occult disease were revealed solely due to resection of the contralateral lobe. This observation is consistent with a large-scale nationwide Chinese study, which demonstrated that patients treated by TT had bilateral disease in 8.2% and occult contralateral disease in 2.4% of cases [23]. They also found a 1.7% contralateral recurrence rate in patients undergoing HT [23]. Two large multicenter retrospective cohorts found bilateral disease in 17 of 306 (5.6%) [27] and 27 of 311 (8.7%) [28] of patients with sMTC, but both did not report whether this was occult disease not preoperatively detected by ultrasound. In summary, the available data for TT to rule out occult bilateral disease in unifocal, nodal negative, small Tumors, appears weak. An important factor to highlight, is that the risk of contralateral foci is much lower in sporadic MTC compared with patients with a germline RET variant (odds ratio 0.043) [29].

Prognostically, our data support existing literature showing that advanced T-stage, lymph node metastases, distant disease, and male sex are significantly associated with worse DFS. These findings are in line with other retrospective analyses [21, 22, 30, 31]. We also confirmed that both vascular and lymphatic invasion are associated with adverse outcomes in sMTC [32–34]. In our cohort, extracapsular extension was the strongest negative prognostic factor, corroborating its well-established role as a marker of aggressive disease [35, 36]. Some histopathological features including desmoplasia can also be assessed intraoperatively by frozen section, facilitating intraoperative decision-making [25].

Interestingly, while high baseline calcitonin levels did not significantly predict recurrence, persistent postoperative calcitonin elevation was clearly associated with inferior outcomes in our cohort. This supports previous findings that postoperative biochemical monitoring is a more reliable predictor of recurrence than baseline values alone, with undetectable postoperative calcitonin strongly associated with long term disease free survival [22, 37–39]. A recent meta-analysis reported that bilateral disease is associated with an increased risk of lymph node metastases, with odds ratios of 3.75 and 2.93 for central and lateral lymph node metastases, respectively [40]. In our cohort, bilateral disease showed only a non-significant trend towards higher rates of lymph node metastasis, which is likely related to the small number of bilateral cases.

Improvements in preoperative imaging, especially high-resolution ultrasound, are enhancing the accuracy of lateralization and risk stratification [19, 20]. As diagnostic certainty increases, so does the feasibility of tailoring surgery to individual risk profiles. In comparison to HT, TT is associated with a higher risk of temporary vocal cord paralysis (4.2% vs. 2.0%), temporary hypoparathyroidism (21.3% vs. 2.2%), and permanent hypoparathyroidism (1.8% vs. 0%) [18]. The short-term impact on health-related quality of life (HRQOL) is higher for TT than HT [41, 42], while long-term data are mixed [43, 44]. Complication rates remain significantly higher in TT compared to HT, despite advances in surgical technique [45, 46]. Where oncologically appropriate, the extent of surgery should be adapted to patients’ individual risk profile to reduce postoperative complications.

Despite a generally low risk of bilateral, and particularly of contralateral disease, improvements in preoperative high-quality ultrasound, and long-term monitoring of calcitonin, data comparing TT and HT for low risk patients are scarce. To date, there are no randomized, controlled trials comparing TT and HT for treatment of sMTC for a selected group of patients with low risk profiles [24].

In the only prospective series to date, a biochemical cure was achieved in 80% of carefully staged non-hereditary cases after HT with systematic central dissection, and no additional benefit from TT [15]. Subsequent retrospective work has reproduced these findings. No significant differences in survival, recurrence-free survival, or biochemical cure were observed between TT and HT in a cohort of 129 patients with unilateral sMTC [21]. The Chinese cohort study of 648 genetically confirmed sMTCs likewise demonstrated equivalent overall survival, structural-recurrence-free survival and biochemical response after propensity matching, even among patients with high-risk features such as elevated calcitonin, nodal metastases or RET M918T mutations [23].

Our study is limited by its retrospective design and small sample size. Although all cases were classified as sporadic based on clinical assessment and family history, not all underwent germline RET testing. However, as the risk for multifocal disease is higher in hereditary MTC [29], the risk of underestimation of contralateral occult disease due to incomplete RET testing is low. The follow-up period, while sufficient to detect early recurrences, may not capture late events.

## Conclusion

The management of medullary thyroid carcinoma remains challenging because the disease can be aggressive and multifocal, yet definitive surgery with TT and neck dissection carries a substantial risk of complications. This study strengthens the evidence that occult contralateral disease in sMTC is rare when high-quality preoperative ultrasound is available. TT may not be necessary for all patients. In well-selected cases—particularly those with unifocal, node-negative T1 tumors without clinically suspect lymph nodes and without histopathological adverse markers, when rigorous calcitonin surveillance is ensured—HT may be oncologically equivalent to TT and associated with lower morbidity. Our data therefore support a paradigm shift toward individualized, risk-adapted surgical management of sMTC. High-quality prospective trials are needed to validate these strategies and refine patient selection.

## Data Availability

The datasets are not publicly available due to patient privacy restrictions but are available from the corresponding author on reasonable request, subject to institutional and GDPR requirements.

## Declarations

### Trial registration

This study was registered in the German Clinical Trials Register (DRKS00037605).
